# The Effect of Antimicrobial Resistance Plasmids Carrying *bla*_CMY-2_ on Biofilm Formation by *Escherichia coli* from the Broiler Production Chain

**DOI:** 10.3390/microorganisms9010104

**Published:** 2021-01-05

**Authors:** Live L. Nesse, Solveig S. Mo, Silje N. Ramstad, Ingun L. Witsø, Camilla Sekse, Anna Eline E. Bruvoll, Anne Margrete Urdahl, Lene K. Vestby

**Affiliations:** 1 Norwegian Veterinary Institute, 0106 Oslo, Norway; solveig.mo@vetinst.no (S.S.M.); silje.ramstad@fhi.no (S.N.R.); camilla.sekse@vetinst.no (C.S.); anna.bruvoll@gmail.com (A.E.E.B.); anne-margrete.urdahl@vetinst.no (A.M.U.); lene.karine.vestby@vetinst.no (L.K.V.); 2 Department of Microbiology, Division of Laboratory Medicine, Oslo University Hospital, 0424 Oslo, Norway; 3 Unit for Food Safety, Faculty of Veterinary Medicine, Norwegian University of Life Sciences, 1432 Ås, Norway; ingun.lund.witso@nmbu.no

**Keywords:** biofilm, *E. coli*, extended-spectrum cephalosporin-resistance, plasmid, bla_CMY-2_, motility

## Abstract

Extended-spectrum cephalosporin-resistant *Escherichia coli* (ESCR *E. coli*) with plasmids carrying the *bla*_CMY-2_ resistance gene have been isolated from the Norwegian broiler production chain through the Norwegian monitoring program for antimicrobial resistance in animals, food and feed, NORM-VET. The aim of the present study was to investigate the biofilm forming abilities of these strains, and in particular to see whether these might be influenced by the carriage of *bla*_CMY-2_ plasmids. The ESCR *E. coli* from the broiler production chain displayed relatively low biofilm forming abilities in the crystal violet biofilm assay as compared to quinolone-resistant *E. coli* (QREC) from the same population (mean ± SD = 0.686 ± 0.686 vs. 1.439 ± 0.933, respectively). Acquisition of two different *bla*_CMY-2_ plasmids by QREC strains reduced their biofilm production in microtiter plates, but not their biofilm production on Congo Red agar plates. Furthermore, motility was reduced, but not planktonic growth. We hypothesize that genes carried by these plasmids may have caused the observed reduction in biofilm formation, possibly mediated through changes in flagellar expression or function. Furthermore, this may help explain the different biofilm forming abilities observed between ESCR *E. coli* and QREC. The results also indicate that the risk of biofilm reservoirs of antimicrobial resistant *E. coli* on in the broiler production is lower for ESCR *E. coli* than for QREC.

## 1. Introduction

In broiler chickens, extended-spectrum cephalosporin-resistant *Escherichia coli* (ESCR *E. coli*) were first described two decades ago [1] and are shown to be globally distributed [2]. This is an increasing cause of concern, as cephalosporins (third, fourth and fifth generation) have been listed by the WHO as critically important antimicrobials in human medicine [3]. In Norwegian broilers, ESCR *E. coli* was detected in 2006 [4]. In the majority of the isolates, the resistance genes are located on plasmids [5,6]. Although the *E. coli* strains carrying these plasmids may not be pathogenic, there is a large potential of dissemination of the resistance genes to pathogens through horizontal transfer of the plasmids. Most isolates carry a *bla*_CMY_ resistance gene, mainly encoded by plasmids belonging to two of the major resistance plasmid families identified in clinically relevant *Enterobacteriaceae*—i.e., IncK and IncI. The plasmids also carry genes that are important for plasmid maintenance and transfer—i.e., plasmid stability systems, transfer associated genes, pilus associated genes, insertion sequence and replication associated genes. In addition, there may be genes encoding other proteins as well as hypothetical proteins [7].

In a screening of all Norwegian broiler chicken flocks during a six-month period in 2016, the overall occurrence of ESCR *E. coli* was 10.3% despite the fact that cephalosporins are not used in Norwegian broiler production [5,6]. Imported parent/grandparent stocks have been shown to be a potential source of ESCR *E. coli*, but investigations have also indicated possible contamination within and between flocks at the same farm [6,8]. In the Norwegian broiler production, the broiler houses are emptied and thoroughly cleaned and disinfected between each flock. Still, the study showed that the odds of a flock being positive for ESCR *Enterobacteriaceae* increased if the previous flock in the same house was positive [6,8]. This indicates that these bacteria may be able to persist in the production environment despite strict hygienic measures.

Formation of bacterial biofilms may be one factor contributing to persistence of ESCR *E. coli* in the production environment. Biofilms are defined as bacterial populations adherent to each other and/or surfaces or interfaces, and enclosed in a self-produced matrix [9,10]. The composition of the matrix differs depending on the species involved in biofilm formation and on the environment, but often consists of proteins, polysaccharides and/or extracellular DNA. Biofilms can accumulate on a wide variety of substrates, and bacteria in biofilms are more tolerant to disinfectants, antimicrobial agents and most other forms of environmental stress, compared to their planktonic counterparts [11,12,13,14,15]. Not surprisingly, biofilms cause problems both in clinical settings, food production facilities and industrial plants.

Large variations in the ability of different *E. coli* strains to produce biofilm have been previously observed in laboratory studies performed under conditions relevant for the food production chain [16,17,18]. Some of these variations have been linked to the presence of curli, which are aggregative amyloid fibers and/or cellulose in the biofilm matrix. Curli and cellulose are known to determine the complex macroscopic architecture of the biofilm [19], contribute to adhesion to surfaces and cell aggregation [20] and might also play a role in resistance to environmental stresses such as desiccation [21] and disinfection [22]. The presence of these components in the matrix can be observed using agar plates containing Congo Red and Coommassie Blue dyes (Congo Red (CR) agar plates) [18,23]. On these plates, production of biofilm can be observed, as well as different biofilm morphotypes that indicate expression of curli and/or cellulose [24,25]. The red, dry and rough (RDAR) morphotype, which has both components, is the most common and the best studied, in both *E. coli* and *Salmonella enterica* [18,24,26].

Previous studies on quinolone-resistant *E. coli* (QREC) in the Norwegian broiler production chain indicated that such strains can form biofilm reservoirs on both inert and organic surfaces in production environments, as well as on meat [16]. In the present study, the biofilm forming abilities of ESCR *E. coli* from the same production chain were characterized to get an indication of their potential to form environmental reservoirs on such surfaces. Furthermore, in most of the ESCR *E. coli* strains from the Norwegian broiler production chain, the antimicrobial resistance genes were encoded on plasmids. Several studies have shown that the acquisition of antibiotic resistance plasmids may be associated with a fitness cost (reviewed by [27]). We therefore also wanted to study whether the presence of the antimicrobial resistance plasmids in the strains influenced their biofilm forming abilities.

## 2. Materials and Methods

### 2.1. Bacterial Strains, Plasmids and Media

The ESCR *E. coli* strains carrying plasmids encoding *bla*_CMY-2_ were originally collected as part of the NORM-VET national monitoring program for antimicrobial resistance in animals, food and feed in 2014 [5], and originated from broiler chicken caecal samples (*n* = 73) and chicken retail meat (*n* = 53). Two previously characterized *bla*_CMY-2_ plasmids from the Norwegian broiler production chain were used in the conjugation experiments (Table 1). The plasmids belong to two different major resistance plasmid families—i.e., IncK and IncI. The QREC recipient strains used in the conjugation experiment were also isolated as part of the NORM-VET program in 2014, and have been previously characterized (Table 1) [18].

All strains were stored at −80 °C in brain heart infusion broth (BHI; Difco, BD, Franklin Lakes, NJ, USA) supplemented with 15% glycerin (Merck KGaA, Darmstadt, Germany) and recovered on blood agar at 37.0 ± 1.0 °C overnight. For overnight cultures, single colonies were transferred into Luria–Bertani broth (LB; Merck KGaA, Darmstadt, Germany), and incubated statically overnight at 37.0 ± 1.0 °C. LB without NaCl (LB^wo^/NaCl; Bacto-tryptone 10 g/L, yeast extract 5 g/L) was used as the test broth in the biofilm assays.

### 2.2. Crystal Violet Biofilm Assay

Biofilm production in microtiter plates was measured in the crystal violet biofilm assay and performed as described previously [16] using 96-well Nunc^TM^ Nunclon^TM^ microtiter plates (Nunc A/S, Roskilde, Denmark) with few modifications. For each strain, 30 μL of an undiluted overnight culture and 100 μL LB ^wo^/NaCl were added in three parallel wells of a microtiter plate. Three wells with 130 μL LB ^wo^/NaCl were used as negative controls on each plate. The microtiter plates were incubated statically for two days at 20.0 ± 1.0 °C or 37.0 ± 1.0 °C. After incubation, optical density at 595 nm (OD_595_) of the planktonic solution was measured as an indication of planktonic growth (Multiscan MS; Thermo Fisher Scientific, Inc., Waltham, MA, USA). Then, the wells were washed with saline, stained with 1% crystal violet solution (Sigma-Aldrich, St. Louis, MO, USA) for 30 min at room temperature, and washed at least three times to remove dye not bound to the biofilm. The biofilm bound dye was dissolved in ethanol-acetone (70:30, *v*/*v*) for 10 min at room temperature before the OD_595_ was measured. The results were calculated by subtracting the median OD_595_ of the three parallel control wells from the median OD_595_ of the three parallel sample wells. The median was used to avoid the influence of accidental outlier data. All samples were tested three times and the mean for each strain was calculated. OD_595_ values higher than mean of the negative controls plus three standard deviations were classified as positive for biofilm production [29]. As the results of the negative control had already been subtracted from the results before this evaluation, the cut-off was set to three SDs of the negative controls. All strains with biofilm formation above the set cut-off were considered positive for biofilm formation in the crystal violet biofilm assay in microtiter plates.

### 2.3. Congo Red Biofilm Assay

**Figure 1:**
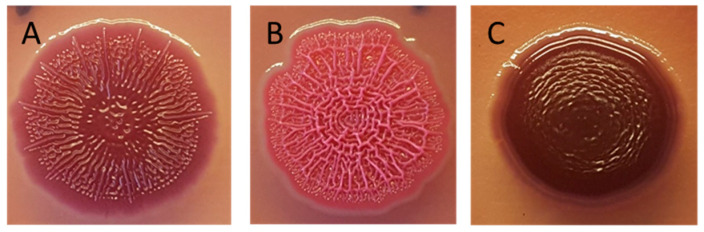
*E. coli* biofilm morphotypes on Congo Red (CR) agar. (**A**) Red, dry and rough (RDAR): indicating expression of curli fimbriae (curli) and cellulose in the biofilm matrix. (**B**) Pink, dry and rough (PDAR): indicating expression of cellulose, but not curli. (**C**) Brown, dry and rough (BDAR): indicating expression of curli, but not cellulose.

Biofilm morphotyping was used to study the differences in matrix components and to measure biofilm formation on an organic surface. The assay was performed as previously described with some modifications [30]. In brief, 1 µL of undiluted overnight culture was inoculated onto Congo Red (CR) agar plates—i.e., LB agar ^wo^/NaCl containing 40 μg/mL Congo Red (Merck KGaA, Darmstadt, Germany) and 20 μg/mL Coommassie brilliant blue (Sigma-Aldrich, St. Louis, MO, USA). After inoculation, the CR agar plates were incubated at 20.0 ± 1.0 °C. All plates were visually examined after two, six and eight days of incubation, and the morphotypes were categorized as: RDAR (red, dry and rough)—indicating presence of curli fimbriae (curli) and cellulose; pink, dry and rough (PDAR)—indicating the expression of cellulose but not curli; BDAR (brown, dry and rough)—indicating expression of curli but not cellulose; and smooth and white (SAW)—indicating no biofilm production (Figure 1). When screening all strains, the assay was performed once. For quantitative evaluation of biofilm formation on CR agar plates, biofilm was incubated at 20.0 ± 1.0 °C or 37.0 ± 1.0 °C, and the diameter of the biofilm was measured after 8 days of incubation. The quantitative assay was performed three times and mean diameter was calculated.

### 2.4. Determination of Phylotypes

All isolates were subjected to phylotyping using multiplex PCR as described previously, with minor modifications [31]. A primer mix was made by mixing 10 µL of a 100 µM stock solution of each of the primers in Table 2 with 20 µL Milli-Q water. The reaction mix was made by adding 2 µL DNA template to 12.5 µL 2× Qiagen Multiplex PCR mix (QIAGEN^®^, Sollentuna, Sweden), 0.5 µL 10 µM primer mix and 10 µL milli-Q water. The cycling conditions used were: (i) 15 min at 95°C; (ii) 30 cycles, with 1 cycle consisting of 30 s at 95 °C, 30 s at 60 °C, and 30 s at 72 °C; (iii) a final extension step of 5 min at 72 °C. The isolates were classified into phylotype A, B1, B2 or D. An isolate belonging to the B2 group, (*E. coli* 2003500827) [32] producing amplicons with all four primer sets, was included as a positive control in each PCR run and the Milli-Q water was used as a negative control.

### 2.5. Conjugation of QREC Strains

Conjugation was performed as previously described [28]. Overnight cultures of recipient and donor strains were mixed together for mating in a 50:1 ratio in 4 mL LB broth, and incubated at 37 °C. From the respective matings, 100 μL broth was plated out on O157 Chrom agar (Difco, Becton Dickinson and company, Sparks, MD, USA) supplemented with 0.5 mg/L cefotaxime and 20 mg/L nalidixic acid, and incubated at 37 °C for 24 h. The chrom agar contained a mix of artificial substrates (chromogens), which release an insoluble colored compound when hydrolyzed by a specific enzyme. Bacteria that utilize different chromogenic substrates will display colonies with different colors. Sampling of the matings was performed as follows: The first samples were collected after four hours of incubation. If no transconjugants were identified, samples were also taken after 24 h of incubation. A real-time PCR with a previously published primer and probe [33] was performed on the transconjugants to confirm conjugative transfer of *bla*_CMY-2_. The strains used as recipients were randomly selected among the QREC strains meeting the following two criteria: (1) OD_595_ > 0.5 in the crystal violet biofilm assay, and (2) pink or turquoise colonies on O157 Chrom agar. The first criterion was to avoid low OD_595_ where the correlation with crystal violet concentration was less reliable, and the second criterion was to differentiate them from the donor strains, which produced blue colonies on the O157 Chrom agar plates. For more information on the recipient strains, see Table 1.

### 2.6. Motility Assay and Microtiter Plate Growth Assay

The motility assay was adapted from Janssens et al. [34] with modifications, and performed using LB with 0.3% agar in 6-well plates (Nunc A/S, Roskilde, Denmark). For each strain, colonies from a blood agar plate were mixed in 5 mL saline to an OD_595_ of 1.0 ± 0.1, and 1 µL of this suspension was inoculated halfway down in the agar in the center of the well. The plates were incubated at 37 °C, and the diameter of the motility zone was measured every hour for the first six hours. The experiment was performed three times for each strain. The motility rate for each experiment was calculated as the mean increase in diameter per hour during the time period between the end of the lag phase and when the fastest strains reached the edge of the well—i.e., between two and six hours of incubation. The final motility rate for each strain was calculated as the mean of the three experiments.

In the microtiter plate growth assay, 5 µL of the same bacterial suspension as used in the motility assay were added to three parallel wells with 195 µL LB broth in a 96-well microtiter plate (Nunc A/S, Roskilde, Denmark). The plates were incubated at 20.0 ± 1.0 °C or 37.0 ± 1.0 °C, and the mean OD_595_ of the parallels measured after 24 h was used as an indicator of bacterial growth at both temperatures. In addition, OD_595_ was measured hourly for the first six hours at 37.0 °C, and mean growth rate of the parallels was calculated as mean increase in OD_595_ per hour in the exponential phase—i.e., between three and six hours of incubation. The experiments were performed three times and the final result for each strain was calculated as the mean of the three experiments.

### 2.7. Statistics

Means were compared using a two-tailed Student’s *t* test or a Mann–Whitney test, depending of the distribution of the data. A chi square test was used to compare frequencies. The Pearson correlation coefficient test was used for correlation calculations. In all tests, *p*-values ≤ 0.05 were considered statistically significant. For all *p*-values ≥ 0.01, the exact values are given with two decimals, whereas lower values are noted as *p* < 0.01.

## 3. Results

### 3.1. Biofilm Production by ESCR E. coli Related to Biofilm Assay and Morphotype

Biofilm formation by the ESCR *E. coli* strains was measured both on the organic surface on CR agar plates and on the inert surface of microtiter plates. In the CR agar plate assay, a qualitative evaluation was made of the presence of biofilm, as well as of the strains’ morphotypes, which indicated presence of cellulose and/or curli in the matrix (Figure 1). In this assay, 96.8% of the strains produced biofilm. Most of the strains displayed the RDAR biofilm morphotype (68.8%), whereas PDAR and BDAR were displayed by 21.6% and 6.4% of the strains, respectively (Table 3).

**Figure 2:**
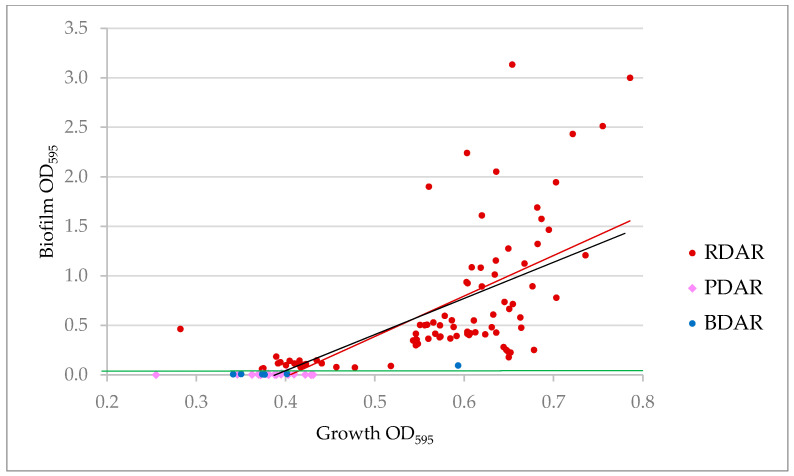
Correlation between OD_595_ indicating growth and OD_595_ indicating biofilm production in the crystal violet biofilm assay for all ESCR *E. coli* strains. Marker color codes show the morphotypes of the strains. Green line indicates cut-off value. The correlation trend line is black for all strains and red for the RDAR strains only. The equation indicates the strength and direction of the relationship and the R^2^ value.

The crystal violet biofilm assay using microtiter plates is a quantitative assay where both biofilm formation and planktonic growth can be measured under the same conditions. In contrast to CR agar plates, only 69.6% of the strains produced biofilm in microtiter plates according to the defined cut-off value for biofilm formation. This group included all the RDAR strains and one of the BDAR strains, and the mean OD_595_ ± SD indicating biofilm production of these was 0.686 ± 0.686. The rest of the BDAR strains and all PDAR and SAW strains did not produce biofilm in this assay (Table 3). Interestingly, the PDAR and BDAR strains displayed significantly lower mean planktonic growth in this assay than the RDAR strains, whereas the nonbiofilm forming SAW strains displayed the same planktonic growth as the RDAR strains (Table 3). A statistically significant correlation was observed between growth and biofilm production of all strains (y = 3.685x − 1.4351, R^2^ = 0.49, *p* < 0.01), as well as for the RDAR strains alone (y = 4.0847x − 1.6545, R^2^ = 0.38, *p* < 0.01) (Figure 2).

### 3.2. Biofilm Production by ESCR E. coli Related to Phylotype

To study the influence of the phylogenetic relationships of the ESCR *E. coli* on biofilm formation, the strains were assigned to one of the four major phylogenetic groups: A, B1, D, or B2. Phylotype D was by far the most common (82.4%), followed by A (14.4%), B1 (2.4%) and B2 (0.8%) Mean biofilm production by strains with phylotype A was significantly higher than strains with phylotype D (*p* < 0.01) (Table 4). Phylotype D had a higher percentage of BDAR, PDAR and SAW strains, which did not produce biofilm in the microtiter plate assay, than phylotype A. We therefore compared biofilm production by the RDAR strains within these two phylotypes. Also in this comparison, the mean biofilm production was significantly higher for phylotype A strains than for phylotype D strains (1.039 ± 1.077 and 0.597 ± 0.499, respectively, *p* = 0.01).

### 3.3. Biofilm Production by ESCR E. coli Related to Source

As the isolates originated from two different sources (ceacum and retail meat), the influence of source on the biofilm forming abilities was studied. No significant difference in the percentage of strains producing biofilm on CR agar plates was observed between strains from caecal and retail meat samples (99.6% and 96.2%, respectively). In the crystal violet biofilm assay, there was a tendency for a higher percentage of RDAR strains, and thereby a higher mean biofilm formation in microtiter plates in the strains from caecal samples, though this was not statistically significant (*p* = 0.22) (Table 5). However, within the group of RDAR strains, there was no difference in mean biofilm formation between strains from caecum and meat (0.701 ± 0.664 vs. 0.678 ± 0.740, *p* = 0.88). Furthermore, no statistical difference was observed between caecal and retail meat samples regarding phylotype distribution, mean biofilm production by the two main phylotypes A and D, or mean biofilm production by the RDAR strains within these phylotypes (Table 5 and Table 6).

### 3.4. Conjugation Experiments with QREC Recipients

To study a possible effect of conjugative transfer of plasmids with ESCR conferring genes on the biofilm forming abilities of QREC strains, twelve QREC strains were separately mated with the donor strains, each carrying a plasmid with the *bla*_CMY-2_ gene (Table 1). One was an IncK plasmid (pNVI1292/IncK), the other an IncI1 plasmid (pNVI2798/IncI1). Transfer of the two plasmids occurred in eight and four mating pairs, respectively, resulting in a total of 12 transconjugants (TCs) (Table 7). All TCs displayed reduced biofilm formation in microtiter plates at 20 °C, and all except one (5792-IncI1) at 37 °C. Consequently, both the TCs with plasmid pNVI1292/IncK (TC-IncK) and those with plasmid pNVI2798/IncI (TC-IncI1) displayed statistically significant reduced mean biofilm production in the crystal violet biofilm assay at both temperatures (Table 8). Reduced motility was also observed for all TCs except one (6035-IncK). The reduction was statistically significant for the combined groups of TCs (mean motility of TCs expressed as a percentage of R = 82.2, *p* < 0.01), and for the group of TC-IncI1. However, the mean reduction for the TC-IncK group was not statistically significant, because the only TC without reduced motility resided in this small group. Biofilm production in the CR agar biofilm assay was not reduced, and all TCs displayed the same morphotype as their respective recipients. Furthermore, no change in planktonic growth was observed in any of the growth assays.

## 4. Discussion

In the present study, acquisition of two plasmids carrying *bla*_CMY-2_ resistance genes [7,28] by QREC strains significantly reduced biofilm production in microtiter plates at both 20 and 37 °C. Earlier observations have shown that acquisition of antibiotic resistance plasmids may be associated with a fitness cost (reviewed by [27]). However, in our study, planktonic growth at the same temperatures was not affected by plasmid acquisition, neither in biofilm stimulating growth medium nor in optimal growth medium. This indicates that the reduced biofilm formation in the tranconjugants was not due to a general fitness cost, but rather a more specific effect by these plasmids. Similar results have been reported for *E. coli* ST 131 isolates after acquisition of plasmids harboring *bla*_CTX-M_, where the transconjugants displayed diminished biofilm formation without a significant defect in growth rate [35]. In another study, curing *E. coli* ST 131 and ST648 isolates of their different *bla*_CTX-M_ carrying plasmids caused some of the isolates to display differences in their biofilm forming abilities [36]. However, in that study, the differences were in both directions, and the results were medium-dependent. Additionally, in said study, no differences in growth curves were observed. The authors suggested that nonresistance genes in some of these plasmids may possess the potential to influence chromosomal gene expression in select strains.

Interestingly, we observed that acquisition of the plasmids also reduced motility in all transconjugants except one (6035-IncK). This fits well with earlier observations of increased motility in *E. coli* strains after being cured from their extended-spectrum cephalosporin-resistance plasmids [36]. A correlation between motility and biofilm formation has previously been reported. Wood et al. found that *E. coli* strains displaying the most vigorous motility as planktonic cells also formed the most robust biofilms in flow cells [37]. Mutations in genes encoding flagellar production and function have resulted in biofilm production defects, indicating that motility is important in initiating biofilm formation (reviewed by [38]). Studies have also shown that flagella may play an important role in surface adhesion [39,40]. Consequently, reduced motility may have been the mechanism through which the plasmids affected biofilm production by the transconjugants in the present study. This is supported by the observation that acquisition of the plasmids did not affect biofilm formation on CR agar plates, indicating that the regulation of biofilm formation was dependent on conditions differing between the two biofilm assays. One such major difference may be whether the bacteria need to be motile to reach the location where biofilm is produced. In the microtiter plate assay, a biofilm formed on the well walls at the liquid–air interface, and it is reasonable to believe that motility may be required for the suspended bacteria to reach this location. This is in contrast to biofilm production in the CR agar plate assay, in which the bacterial suspension was placed directly on top of the agar surface where the biofilm formed, and motility may thus be of minor importance. This assumption is supported by several previous reports. In a recent study, two *Salmonella* flagellar mutants were reported to be defective in the early stage of biofilm formation in microtiter plates in the crystal violet biofilm assay [41]. Another study on *Salmonella* showed that flagella were not required for the production of RDAR biofilms on CR-agar plates, whereas absence of flagella did influence biofilm formation on air–liquid, surface–liquid or cell–cell–liquid interfaces [42]. Accordingly, we have observed that two nonmotile *Salmonella* (*S*.) *enterica* Δ*fliA*-mutants, which produced little or no biofilm in microtiter plates, were still able to form biofilm on CR agar plates (unpublished results). This was in contrast to their wild-type counterparts (*S. enterica* serovar Typhimurium ATCC14028 and *S*. *enterica* serovar Agona 2168-10), which were strong biofilm formers in both assays [43]. Furthermore, results from a study on *E. coli* suggested that flagella did play an architectural role of in *E. coli* biofilms on agar plates, but this was not related to motility [44].

Based on the results, we hypothesize that the observed reduction in biofilm formation after acquisition of the plasmids was due to specific regulation by plasmids. Two possible candidate genes to be involved in such a regulation are carried by the plasmids used in our conjugation studies—i.e., *yqiK* and *sugE*. The YqiK protein is suggested to be implicated in stress sensing in *E.coli*. A *yqiK*-deficient mutant strain showed enhanced swimming behavior, implying that this gene downregulates motility [41]. Regarding the other candidate, *sugE*, a deletion of this gene in *Klebsiella pneumonia* provided a 1.5-fold enhanced ability to produce biofilm, suggesting a biofilm repressing function of the gene [42]. However, the plasmids also carry a number of genes encoding proteins of unknown function, which potentially may also be involved in such regulation, and further studies are needed to identify the gene or genes in question.

The ESCR *E. coli* strains from the broiler production chain screened in the present study all carried plasmid-encoded *bla*_CMY-2_ genes. Consequently, the observed effect of the plasmids on biofilm formation may contribute to explain why these strains displayed lower mean biofilm productions in the crystal violet biofilm assay than that of 158 QREC from the same broiler population tested earlier [18]. None of those QREC strains had MIC (minimum inhibitory concentration) profiles corresponding to the possible presence of plasmid-mediated resistance genes [5]. Only 69.6% of the ESCR *E. coli* strains produced biofilm in microtiter plates, in contrast to 84.2% of the QREC strains, according to defined cut-off values for positive biofilm production. Although there were large variations in biofilm formation within both groups, the mean OD_595_ of the biofilm positive strains was twice as high for the QREC strains as compared to the ESCR *E. coli* strains (mean ± SD = 1.439 ± 0.933 vs. 0.686 ± 0.686, respectively). For both the ESCR *E. coli* and the QREC strains, RDAR was the most common morphotype (68.8% and 69.6%, respectively) and the RDAR strains were in general the best biofilm producers. However, within all morphotypes, the ESCR *E. coli* strains were substantially poorer biofilm formers in microtiter plates. Strong biofilm producing abilities in microtiter plates have previously been shown to correlate with long-term persistence in various production environments [25,43]. Consequently, these results may indicate that the risk of biofilm reservoirs in the broiler production chain environment may be lower for ESCR *E. coli* than for QREC.

Both in the ESCR *E. coli* strains and the previously tested QREC strains, biofilm formation in microtiter plates seemed to be strongly influenced by the presence of curli and cellulose in the biofilm matrix as indicated by the morphotype [18]. As curli are reported to be involved in adhesion to surfaces and cell aggregation [44], and cellulose is suggested to play a role in the adhesion to at least some surfaces [18], it is reasonable that the presence of these matrix components may influence biofilm formation. However, in the present study we also observed the levels of planktonic growth of the ESCR *E. coli* strains during biofilm formation in the microtiter plates and found that these levels were positively correlated to the amount of biofilm produced. Thus, we do not know the exact underlying cause of the observed lack of biofilm formation in the PDAR and BDAR strains, as it could be explained by a missing matrix component, a low level of planktonic growth, or a combination of these two factors. However, these results clearly indicate that level of planktonic growth is an important factor contributing to biofilm formation under these conditions. Interestingly, the four strains that did not produce biofilm in any of our assays displayed the same level of planktonic growth as the RDAR strains. This may indicate that in these specific strains, the observed lack of biofilm production is more likely due to a defect in genes involved in such production [18,24,26].

Interestingly, 96.8% of the ESCR *E. coli* strains produced biofilm on CR agar plates, including those with very low or even no biofilm formation in microtiter plates. Thus, it appears to be “easier” for the strains to form biofilm on these agar plates than in microtiter plates. Whether this can be explained by the differences between the two tests regarding type of surface, nutrient availability, application method, motility requirements and/or other factors, is not known.

In the present study, a significant difference in biofilm production in microtiter plates of the ESCR *E. coli* strains was also observed between strains of the phylotypes A and D, which could not be explained by differences in morphotype. The results support earlier observations of association between biofilm forming abilities and phylotype/serotype/serovar [16,18,25]. Phylotype D was displayed by the majority of the ESCR *E. coli* strains, while B2 was the most common in the QREC strains earlier studied. The QREC strains were substantially better biofilm formers than the ESCR *E. coli* strains when comparing within phylotypes. The observed difference in mean biofilm production by ESCR *E. coli* strains and QREC strains were therefore not due to the different phylotype distribution in these two groups of strains.

## 5. Conclusions

The results from the present study showed that acquisition of *bla*_CMY-2_ plasmids by QREC strains reduced their biofilm forming abilities in microtiter plates. This did not appear to be due to a general fitness reduction, as planktonic growth was not affected. We therefore hypothesize that genes encoded on these plasmids may have caused the observed reduction in biofilm formation. Furthermore, we suggest that this effect may have been mediated through changes in flagellar expression or function, as acquisition of the plasmids also resulted in reduced motility.

This effect may explain why ESCR *E. coli* strains carrying *bla*_CMY-2_ plasmids from the broiler production displayed substantially lower biofilm forming abilities than QREC from the same population. A practical consequence of this observed difference in biofilm forming abilities may be that the risk of biofilm reservoirs being produced in the broiler production chain is lower for ESCR *E. coli* than for QREC.

## Figures and Tables

**Table 1:** Information on strains and resistance plasmids used in the conjugation studies.

Strain No	Source	Phylo-Type	Morpho-Type	Plasmids	Plasmid Size	Ref.
**Donor strains**						
2012-01-1292	Retail meat	D	RDAR	pNVI1292/IncK (accession no KU312044)	79.3 kB	[7]
2012-01-2798	Retail meat	A	RDAR	pNVI2798/IncI1	100–110 kB	[28]
**Recipient strains**						
2014-01-5835	Retail meat	A	RDAR			[18]
2014-01-11	Retail meat	B1	RDAR			[18]
2014-01-1268	Caecum	B1	RDAR			[18]
2014-01-1499	Retail meat	B1	RDAR			[18]
2014-01-5572	Retail meat	B1	RDAR			[18]
2014-01-6035	Retail meat	B1	RDAR			[18]
2014-01-7132	Retail meat	B1	RDAR			[18]
2014-01-4271	Caecum	D	RDAR			[18]
2014-01-5565	Caecum	D	RDAR			[18]
2014-01-5792	Caecum	D	RDAR			[18]
2014-01-6041	Caecum	D	RDAR			[18]
2014-01-6123	Caecum	D	RDAR			[18]

**Table 2:** Primers used for determination of phylotypes.

Primer	Sequence	Size
gadA.F	5′-GATGAAATGGCGTTGGCGCAAG-3′	373 bp
gadA.R	5′-GGCGGAAGTCCCAGACGATATCC-3′	
chuA.F	5′-ATGATCATCGCGGCGTGCTG-3′	281 bp
chuA.R	5′-AAACGCGCTCGCGCCTAAT-3′	
yjaA.F	5′-TGTTCGCGATCTTGAAAGCAAACGT-3′	216 bp
yjaA.R	5′-ACCTGTGACAAACCGCCCTCA-3′	
TSPE4.C2 F	5′-GCGGGTGAGACAGAAACGCG-3′	152 bp
TSPE4.C2 R	5′-TTGTCGTGAGTTGCGAACCCG-3′	

**Table 3:** Biofilm production and planktonic growth by extended-spectrum cephalosporin-resistant (ESCR) *E. coli* strains, as indicated by OD_595_ in the crystal violet biofilm assay, within each morphotype and in the total material.

			Biofilm			Growth		
Morpho-Type	No	%	Mean OD_595_ ± SD	Min. OD_595_	Max. OD_595_	Mean OD_595_ ± SD	Min. OD_595_	Max. OD_595_
RDAR	86	68.8	0.693 ^A^ ± 0.687	0.062	3.134	0.575 ^A^ ± 0.104	0.282	0.786
BDAR	8	6.4	0.018 ^B^ ± 0.032	0.000	0.096	0.388 ^B^ ± 0.087	0.316	0.593
PDAR	27	21.6	0.005 ^B^ ± 0.006	−0.006	0.026	0.386 ^B^ ± 0.033	0.255	0.431
SAW	4	3.2	0.006 ^B^ ± 0.005	0.003	0.013	0.500 ^A^ ± 0.082	0.389	0.584
TOTAL	125	100.0	0.479 ± 0.652	−0.006	3.134	0.520 ± 0.124	0.255	0.786

SD = standard deviation, Min. = minimum, and Max. = maximum. ^A^, ^B^: means with same letter in same column are not statistically different (*p* > 0.05).

**Table 4:** Biofilm production by ESCR *E. coli* strains as indicated by OD_595_ in the crystal violet biofilm assay, as well as distribution of morphotypes within each phylotype.

		Biofilm			% of Each Morphotype			
Phylo-Type	No	Mean OD_595_ ± SD	Min. OD_595_	Max OD_595_	RDAR	BDAR	PDAR	SAW
A	18	0.924 ^A^ ± 1.066	0.003	3.134	88.9	0.0	0.0	11.1
B1	3	0.982 * ± 1.327	0.179	2.513	100.0	0.0	0.0	0.0
B2	1	0.096 *	-	-	100.0	0.0	0.0	0.0
D	103	0.391 ^B^ ± 0.491	−0.006	2.433	65.0	6.8	26.2	1.9

SD = standard deviation. ^A^, ^B^: means with different letters are statistically different (*p* ≤ 0.05). * mean has not been statistically compared to others due to the low number of strains.

**Table 5:** Biofilm production by ESCR *E. coli* strains as indicated by OD_595_ in the crystal violet biofilm assay, as well as distribution of morphotypes within each phylotype.

		Biofilm	% of Each Morphotype				% of Each Phylotype			
Source	No	Mean OD_595_ ± SD	RDAR	BDAR	PDAR	SAW	A	B1	B2	D
Caecum	73	0.540 ± 0.650	76.7	4.1	17.8	1.4	15.1	1.4	1.4	82.2
Meat	52	0.393 ± 0.651	57.7	9.6	26.9	5.8	13.5	3.8	0.0	82.7

SD = standard deviation.

**Table 6:** Comparison of biofilm production by ESCR *E. coli* strains from chicken caecal and retail meat samples, as indicated by OD_595_ in the crystal violet biofilm assay, within phylotypes A and D of all strains, and of strains with the morphotype RDAR.

		Chicken Caecal Samples		Chicken Retail Meat Samples		
	Phylo-Type	No	Mean Biofilm OD_595_ ± SD	No	Mean Biofilm OD_595_ ± SD	*p*-Value *
All strains	A	11	1.076 ± 1.066	7	0.686 ± 1.104	0.47
	D	60	0.417 ± 0.430	43	0.354 ± 0.569	0.53
RDAR strains	A	11	1.076 ± 1.066	5	0.959 ± 1.225	0.85
	D	45	0.566 ± 0.410	23	0.657 ± 0.641	0.48

SD = standard deviation. * Mean OD_595_ of caecal samples vs. retail meat samples.

**Table 7:** Transconjugant strains obtained.

Recipient Quinolone-Resistant *E. coli* (QREC) Strains	Transconjugant Strains	
	With Plasmid pNVI1292/IncK	With Plasmid pNVI2798/IncI1
2014-01-5835	-	-
2014-01-11	11-IncK	11-IncI1
2014-01-1268	1268-IncK	-
2014-01-1499	-	-
2014-01-5572	5572-IncK	5572-IncI1
2014-01-6035	6035-IncK	6035-IncI1
2014-01-7132	-	7132-IncI1
2014-01-4271	-	-
2014-01-5565	-	5565-IncI1
2014-01-5792	-	5792-IncI1
2014-01-6041	-	6041-IncI1
2014-01-6123	-	6123-IncI1

**Table 8:** Mean relative levels of biofilm production, motility and growth by each transconjugant (TC) as compared to their respective recipient (R), expressed as % of R. Means of all TCs with the same plasmid are highlighted with bold numbers. Statistically significant differences between TCs and Rs (*p* < 0.05) are highlighted with a grey background.

Parameter	Biofilm				Motility	Growth				Growth Rate
Assay	CVBA	CVBA	CR-BA	CR-BA	MA	CVBA	CVBA	MGA	MGA	MGA
Temperature	20 °C	37 °C	20 °C	37 °C	37 °C	20 °C	37 °C	20 °C	37 °C	37 °C
**TC-IncK**										
11-IncK	77.7	75.3	85.7	100.0	81.0	94.5	97.8	98.0	98.0	98.7
1268-IncK	68.6	41.6	83.3	100.0	82.4	81.0	136.1	101.4	105.9	102.8
5572-IncK	76.6	75.4	150.0	120.0	66.7	91.4	98.3	100.2	99.4	99.3
6035-IncK	92.8	54.1	100.0	133.3	110.0	96.6	98.5	101.4	99.4	102.2
**Mean**	**78.9**	**61.6**	**104.8**	**113.3**	**85.0**	**90.9**	**107.7**	**100.2**	**100.7**	**100.7**
*p*-value *	0.02	0.03	0.89	0.22	0.15	0.11	0.45	0.78	0.71	0.53
**TC-IncI1**										
11-IncI1	71.3	83.5	85.7	90.0	69.0	96.1	97.6	100.4	94.0	96.8
5565-IncI1	60.9	65.2	93.8	81.3	82.2	102.2	100.0	99.2	103.1	99.5
5572-IncI1	65.4	93.3	120.0	120.0	63.0	91.7	101.1	100.2	98.7	96.7
5792-IncI1	91.6	113.6	86.7	100.0	83.6	111.1	109.4	97.4	98.5	97.5
6035-IncI1	67.5	72.8	100.0	108.3	75.9	92.5	89.0	100.8	98.2	97.7
6041-IncI1	55.1	29.2	82.4	92.3	96.4	96.1	89.9	100.0	96.8	102.3
6123-IncI1	96.6	89.7	100.0	93.8	86.0	117.7	105.9	97.4	102.4	99.4
7132-IncI1	81.0	52.6	100.0	73.3	83.3	109.6	105.5	100.6	97.1	96.9
**Mean**	**73.7**	**75.0**	**96.1**	**94.9**	**79.9**	**102.1**	**99.8**	**99.5**	**98.6**	**98.4**
*p*-value *	0.03	0.02	0.22	0.25	<0.01	0.48	0.24	0.34	0.21	0.17

* Mean of recipients vs. mean of transconjugants, two-tailed paired Student’s *t* test. Temp. = incubation temperature, TC-IncK = transconjugants with plasmid pNVI1292/IncK, TC-IncI1 = transconjugants with plasmid pNVI2798/IncI1, CVBA = crystal violet biofilm assay, CR-BA = CR agar biofilm assay, MA = motility assay, and MGA = microtiter plate growth assay.

## Data Availability

The datasets used and/or analysed during the current study are available from the corresponding author on reasonable request.
